# Detection of new pathways involved in the acceptance and the utilisation of a plant-based diet in isogenic lines of rainbow trout fry

**DOI:** 10.1371/journal.pone.0201462

**Published:** 2018-07-31

**Authors:** Thérèse Callet, Mathilde Dupont-Nivet, Marianne Cluzeaud, Florence Jaffrezic, Denis Laloë, Thierry Kerneis, Laurent Labbé, Edwige Quillet, Inge Geurden, David Mazurais, Sandrine Skiba-Cassy, Françoise Médale

**Affiliations:** 1 UMR GABI, INRA, AgroParisTech, Université Paris-Saclay, Jouy-en-Josas, France; 2 UMR NuMéA, INRA, St-Pée-sur-Nivelle, France; 3 PEIMA, INRA, Sizun, France; 4 IFREMER, Brest, France; National Cheng Kung University, TAIWAN

## Abstract

To meet the growing demand of fish feed for aquaculture, an increasing proportion of marine ingredients are being replaced by blends of plant products. However, the total replacement of marine ingredients in salmonid diets impairs fish performance. This is particularly true during the early fry stage and this stage is therefore considered of particular importance. In rainbow trout (RBT), the existence of a genetic variability to survive and grow with plant-based diets devoid of marine ingredients has now been proved, but the mechanisms behind are little studied especially at early stage. To investigate these, we analysed the whole transcriptome of three isogenic lines of RBT fry, which have similar growth when fed a marine resources-based diet (M diet) but which highly differ in their responses to a plant-based diet (V diet). Analysis of transcriptomes profiles revealed 1740, 1834 and 246 probes differentially expressed among the three genotypes when fed the V diet. The use of these lines led to the discovery of potential molecular markers linked to plant-based diet utilisation, some of them belonging to new pathways, never described before. An important number of genes was related to immunity, but further investigations are needed to better understand the difference between the genotypes in their immune status response to V diet exposure. Finally, differences in expression of genes related to feed intake and sensory perception among genotypes suggested that the mechanisms underlying the differences in growth on plant-based diet are closely linked to diet acceptance. Research on plants components affecting feed intake should be thus further explored.

## Introduction

Fish consumption per person per year has almost doubled over the last 50 years, growing from 9.9 kg in 1960 to 20 kg in 2014, and is expected to reach 22.4 kg in 2022 [1]. Aquaculture has grown exponentially since 1950 to meet fish demand, thereby increasing the demand for feedstuffs. The ingredients traditionally used in aquafeeds were mostly based on fishery-derived products. Availability of these ingredients is however limited due to the regulation by fishing quotas and cannot meet the increasing demand [2]. To address human protein needs and the increasing demand that is placed on fish supply, aquaculture needs to further expand, while at the same time it must reduce its reliance on marine by-products [2, 3]. Over the years, researchers and the feed industry have worked to replace marine ingredients (fish oil and fishmeal) by plant-derived ingredients in fish feeds, especially for carnivorous species, such as salmonids which are large consumers of fish oil (FO) and fishmeal (FM). This research has resulted in a remarkable increase in the level of plant products used, coupled with an improved diet formulation [4]. A replacement threshold is, however, met since growth performance and survival rate of salmonids, such as rainbow trout (RBT), are impacted when a diet contains more than 80% of plant products [5, 6]. Different hypotheses have been proposed to explain these decreased performances.

Firstly, altered performance observed with highly substituted plant-based diets could be partly explained by the reduction of feed intake [7]. Secondly, even with a diet composition carefully formulated to meet the all known nutrient requirements of RBT, plant-based diets still differ from a marine resources-based diet in their amino acids (AA), fatty acids (FA), and carbohydrates profiles, in their mineral contents, and often by the presence of anti-nutritional factors [8]. Numerous nutrigenomic analyses have been conducted to investigate how these differences affect fish metabolism. They mainly focused on the liver [5, 9–15] and the intestines [13, 16], key organs in nutrition. Although, results from these studies depend on the species studied, replacement rate and diet composition, the expression of several key genes involved in metabolism was found to be particularly sensitive to either vegetable meal and/or vegetable oils. This is especially true for genes coding for enzymes that promote biosynthesis of omega-3 long-chain fatty acids (LC-PUFA) and cholesterol, as both omega-3 LC-PUFA and cholesterol are lacking in vegetable oils [5, 9–15]. Genes related to the utilisation of glucose (glycolysis, glycogenogenesis) and production of endogenous glucose (glycogenolysis, gluconeogenesis) are impacted likely because of a change in starch content of diets with plant-ingredients [5, 9, 13, 14]. Misregulation of certain amino acid pathways and stimulation of proteolysis [5, 14] are also commonly observed.

Finally, those transcriptomic analyses have also revealed that expression of genes from both innate and adaptive immunity are often disturbed [5, 13, 14], but implications for fish immune responses and how these changes could impact growth rate are not yet clear.

Almost all the previously cited transcriptomic analyses investigated the effect of the diet, and very few considered the impact of fish’s genetic background. It has, however, been shown that a high variability exists in the growth responses among RBT individuals from a same population when fed a 100% plant-based diet. Significant genotype × diet interactions associated with body weight [17, 18], feed intake and feed efficiency [19] were also reported in RBT. Together, these results suggest that some genotypes are more able than others to survive and grow on a plant-based diet, highlighting the potential to improve growth rate by exploiting genetic variability. Morais *et al.* described the changes in the liver [11, 12] and intestines [16] of two post-smolt Atlantic salmon groups with contrasting phenotypes (lean or fat), that were fed vegetable oils. A study on European sea bass revealed differences between two families characterized by their different abilities to use a plant-based diet [14]. And only two studies focused on RBT, and analysed two families with different ability to grow with a diet devoid of fishmeal [20, 21].

The previous cited studies have investigated the effect of the fish genotype on plant-based diets utilization on later stages only. However, the weeks immediately after start-feeding have been also identified as a critical step in the utilization to such diets, as this period show the highest genotype×diet interaction for RBT mean body weight and length [18] and a significant decrease in fish survival rate [22, 23]. Moreover, previous studies have shown that an early exposure to a 100% plant-based diet improves later fish performance when fed again the same extreme diet [24], and identified different molecular markers linked to plant-based diet adaptation [25]. The molecular mechanisms underlying this genetic variability during the first few weeks after the first-feeding, stage of particular importance, have thus not been investigated.

The originality of the present study was to identify genes contributing to the differential ability of RBT to use plant-based diets from very first feeding. The second aim was to focus on early stage, which is a highly critical step, thus transcriptomic analyses were carried out 5 weeks after the first feeding on whole fry as fish are too small at this stage to analyse accuratly specific organs.

We studied three isogenic lines generated for scientific research [26], which exhibit similar growth rates when fed a marine resources-based diet, but different growth rates when fed a plant-based diet after long term feeding trials, explained by differences in feed efficiency and feed intake [19, 24]. Because of the within line genetic homogeneity, a limited within line (residual) phenotypic variability is expected. The specific features of these three isogenic lines, along with the use of an extreme plant-based diet to emphasize the differences between the genotypes, are expected to enhance the power and relevance of the analyses to detect potential molecular markers linked to plant-based diet utilisation after a short-term period at start-feeding.

## Materials and methods

### Animal material

The three heterozygous isogenic lines studied, denominated R23h, AB1h and A22h, were produced at PEIMA facilities (INRA, Sizun, France). They were obtained by mating all homozygous females from one single isogenic line with individual males from three other homozygous isogenic lines. The homozygous lines were obtained after two generations of gynogenesis and further maintained by within line pair-mating [26]. In order to have enough fish for the whole experience, ova were collected from 33 different females from the same isogenic line (thus sharing the same genome), and were then mixed up. Differences observed between genotypes could then be attributed only to paternal effects.

### Experimental diets and feeding trial

Two experimental diets with different levels of FM and FO or plant ingredients were formulated and manufactured in INRA facilities (INRA, Donzacq, France). Composition of both diets, along with the FA and the AA profiles, are presented in Tables 1 and 2. Diets were formulated to contain similar levels of protein, energy, and lipid. The control M diet contained fishmeal, fish oil and whole wheat as major ingredients (M diet). The plant-based diet (V diet) was devoid of marine ingredients, and contained a blend of vegetable oils (rapeseed, palm and linseed oil) and vegetable meals (faba bean, corn, soybean, wheat gluten, peas, and white lupin) with added L-Lysine and L-methionine to meet the nutrient requirements of RBT. The V diet also contained an attractant mix. Both diets were extruded in order to reduce antinutritional factors.

**Table 1:** Ingredients and proximal composition of the experimental V and M diets (DM: Dry matter).

	V diet	M diet
**Ingredients (%)**		
**Fishmeal** (Southern hemisphere, Sopropêche, France)	**0**	**65**
Extruded whole wheat (SudOuest Aliment, France)	4	21
Fava bean (CP)	10	0
Corn gluten (CP 60; Inzo, France)	17	0
Wheat gluten (CP 70; Roquette, France)	17	0
Soybean meal (CP 48; Inzo, France)	12	0
White lupin seed meal (Terrena, France)	5	0
Extruded peas (Aquatex, Sotexpro, France)	12.5	0
**Fish oil** (Southern hemisphere, Sopropêche, France)	**0**	**11**
Rapeseed oil (Daudruy, France)	6	0
Linseed oil (Daudruy, France)	3.6	0
Palm oil (Daudruy, France)	2.4	0
Soy-lecithin (Louis François, France)	2	0
L-Lysine (Eurolysine)	0.5	0
L-Methionine (Evonik, Germany)	0.5	0
CaHPO4.2H20 (18%P; 22%Ca)	3	0
Min. and Vit. premix, INRA^a^	3	3
Attractant mix^b^	1.5	0
**Composition (% DM)**		
Dry matter	96.9	97.6
Crude protein	51.4	50.1
Crude fat	18.5	19.4
Starch	9.6	14.1
Ash	6.5	12.7
Energy (kJ/g DM)	23.6	22.6

*^a^*Mineral premix (g or mg kg-1 diet): calcium carbonate (40% Ca), 2.15 g; magnesium oxide (60%Mg), 1.24 g; ferric citrate, 0.2 g; potassium iodide (75%I), 0.4 mg; zinc sulphate (36%Zn), 0.4 g; copper sulphate (25%Cu), 0.3 g; manganese sulphate (33%Mn), 0.3 g; dibasic calcium phosphate (20%Ca, 18%P), 5 g; cobalt sulphate, 2 mg; sodium selenite (30%Se), 3 mg; KCl, 0.9 g; NaCl, 0.4 g (UPAE, INRA); And Vitamin premix (IU or mg kg-1 diet): DL-a tocopherol acetate, 60 IU; sodium menadione bisulphate, 5 mg; retinyl acetate, 15,000 IU; DL-cholecalciferol, 3,000 IU; thiamin, 15 mg; riboflavin, 30 mg; pyridoxine, 15 mg; B12, 0.05 mg; nicotinic acid, 175 mg; folic acid, 500 mg; inositol, 1,000 mg; biotin, 2.5 mg; calcium pantothenate, 50 mg; choline chloride, 2,000 mg (UPAE, INRA).

*^b^*Attractant mix: glucosamine, 0.5 g; taurine, 0.3 g; betaine, 0.3 g; glycine, 0.2 g; alanine, 0.2 g.

**Table 2:** Fatty acids and amino acids profiles of the experimental V and M diets (DM: Dry matter).

	V diet	M diet
**Fatty acid composition (% of total fatty acid)**		
**Saturated**	19.8	39.2
**MUFA**	39.0	29.5
**n-6 PUFA**	24.0	4.7
18:2 n-6 (LA)	24.0	3.2
**n-3 PUFA**	17.1	19.1
18:3 n-3 (ALA)	17.1	1.1
20:5 n-3 (EPA)	0.0	9.5
22:6 n-3 (DHA)	0.0	5.2
**Amino acid composition (% DM)**		
Aspartic acid (Asp)	3.5	3.9
Glutamic acid (Glu)	11.2	6.3
Alanine (Ala)	2.4	2.8
Arginine (Arg)	2.7	2.6
Cysteine (Cys)	0.7	0.4
Glycine (Gly)	1.8	2.8
Histidine (His)	1.0	1.0
Isoleucine (Ile)	2.0	2.0
Leucine (Leu)	4.3	3.3
Lysine (Lys)	2.2	3.3
Methionine (Met)	1.2	1.2
Phenylalanine (Phe)	2.4	1.9
Proline (Pro)	3.4	1.7
Serine (Ser)	2.1	1.7
Threonine (Thr)	1.5	1.9
Tyrosine (Tyr)	1.6	1.2
Valine (Val)	2.2	2.4

The V diet was devoid of 20.5 n-6 and 22.6 n-3. It was lower in saturated FA and higher in MUFA, 18:2 n-6 and 18:3 n-3 than the M diet. The two diets also differ in amino acids profiles, especially in glutamic acid content which was twice higher in the V-diet than in the M-diet.

Before the first feeding, the fry were randomly distributed into 18 tanks (0.25m^3^) and supplied with natural spring water at a constant temperature of 11.4°C (6 tanks/line with on average 350 fish), and under artificial photoperiod condition (from 8am to 8pm). At this stage, no difference in fry mean body weights were detected. At 42 days post fertilization (dpf), fish received their first feeding. For each line, three tanks received the control diet (M diet) and three tanks received the V diet. Feed was distributed *ad libitum* during 5 weeks with automatic feeders over 8h of the lighting period.

After the first 5 weeks of feeding (74 dpf), total fish weight of each tank was measured, the number of fish was counted and mean body weight of fry were calculated. Whole body fry were sampled, anaesthetized and then euthanized (anesthetic overdose) 8h after their last meal (n = 6/condition: genotype×diet). All the samples were then immediately frozen in liquid nitrogen and stored at -80°C until further analysis.

### RNA extraction

Total RNA was extracted from 6 individual fry per treatment, using the TRIzol reagent method (Invitrogen, Carlsbad, CA, USA), according to the manufacturer’s recommendations. The concentration of extracted RNA was analysed using a spectrophotometer (ND-1000, NanoDrop) by measuring absorbance at 260nm. Quality of RNAs was checked with Bioanalyzer (Agilent Technologies, Kista, Sweden).

### Microarrays, cDNA labelling and hybridisation

RNA samples from fry from the three isogenic lines after 5 weeks of feeding with either the M diet or the V diet were analysed with microarray technology. Microarray analyses were performed on an Agilent-based microarray platform with 8 X 60 K probes per slide. This platform is based on a high density rainbow trout oligonucleotide microarray resource, which has been enriched with oligonucleotides utilizing recent NGS data from rainbow trout [27](Agilent Technologies, Massy, France; Gene Expression Omnibus (GEO) accession no. GPL15840). The platform has been employed and validated in several previous studies [25, 28].

For each sample, 150ng of total RNA was amplified and labelled using Cy3-CTP according to the manufacturer’s instructions (Agilent). cRNA were obtained by first reverse transcribed RNA, using a polyDT T7 primer (denaturation step: 10min at 65°C, reaction step: 2hr at 40°C, inactivation step: 5min at 70°C). cRNA were then labelled with Cy3-dye (2hr at 40°C). Excess dye was removed using a RNeasy kit (Qiagen). The level of dye incorporation was evaluated using a spectrophotometer (Nanodrop ND1000, LabTech) (Yield>0.825*μ*g cRNA and specific activity>6pmol of Cy3 per *μ*g of cRNA). 600ng of Cy3-cRNA was then fragmented with a specific buffer (30 minutes at 60°C) and hybridised on a sub-array. Hybridisation was performed in a microarray hybridisation oven (Agilent) for 17h at 65°C. After a washing step, slides were scanned (Agilent DNA Microarray Scanner, Agilent Technologies, Massy, France) using the standard parameters for a gene expression 8x60K oligoarray (3*μ*m and 20bits). Data were then obtained with the Agilent Feature Extraction software (10.7.1.1). Raw data are available in NCBI’s GEO database (accession number: GSE92673).

### Quantitative real-time PCR analysis

In order to confirm the microarray results, the same RNA samples as previously used for the microarray analysis were used to determine relative gene expression levels by quantitative real-time PCR (qPCR) of 9 genes selected among those found differentially expressed. One *μ*g of total RNA was reverse-transcribed to cDNA with SuperScript III RNase H reverse transcriptase (Invitrogen, Carlsbad, CA, USA) using random dT Primers (n = 6/condition). QPCR were performed using the Roche Lightcycler 480 system (Roche Diagnostics, Neuilly-sur-Seine, France). Two *μ*L of diluted cDNA were mixed with 3 *μ*L of Light cycler 480 SYBR Green I Master mix, 0.12 *μ*L of each primer (400 nM) and 0.76 *μ*L DNase/RNase-free water (5 Prime GmbH, Hamburg, Germany). Forward and reverse primers were used at a final concentration of 400nM. Primers were designed with Primer 3 software (Supplementary File 1). Thermal cycling was initiated with the incubation at 95°C for 10 min. Forty five steps of PCR were performed, each one consisting of heating at 95°C for 15 sec for denaturing, and at 60°C for 10 sec for annealing and a third extension step at 72°C for 15 sec. Melting curves were systematically monitored (with a gradient of 0.5°C/10 sec from 55°C to 94°C). Samples without reverse transcriptase and samples without RNA were run for each reaction as negative controls. Primers sequences, along with their efficiencies are presented in the Supplementary file 1.

### Statistical analysis

All the statistical analyses were performed using the R-software (version 3.2.5) [29] and associated Bioconductor packages. For mean body weights calculated at 74 dpf, the normality and homogeneity of residuals were tested with the Shapiro-Wilk’s test and the Bartlett test, respectively. A two-way analysis of variance (ANOVA) was performed to assess effects of genotype, diet, and genotype×diet interaction, followed by a Tukey’s range test.

Data from the microarray analysis were transformed with a logarithmic transformation and scale-normalised. The quality of each sample was controlled using the ArrayQualityMetrics package [30], and two samples were excluded from the analysis, as they did not pass the quality control. Data were analysed using the package Limma [31] and for each comparison Limma t-tests were performed. A Benjamini-Hochberg correction for multiple tests was applied and an adjusted P-value cutoff = 0.05 was considered.

**Fig 1:**
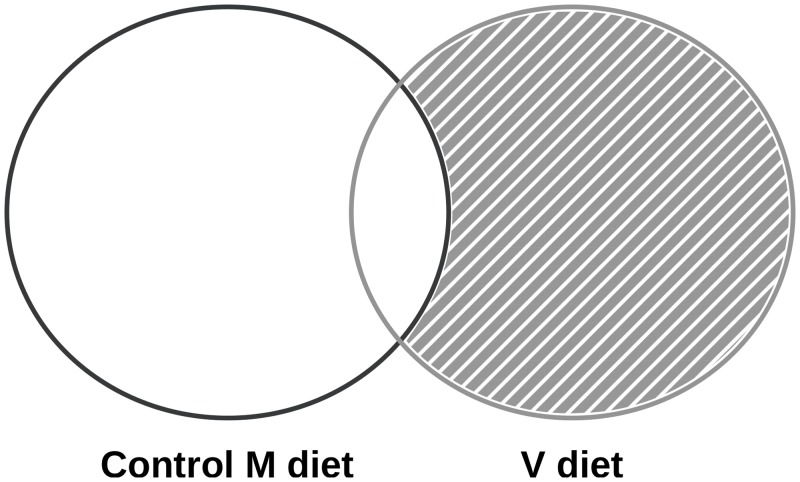
Transcriptomic analysis strategy. Venn diagrams show the numbers of differentially expressed probes (Adjusted P-value < 0.5) when compared the three isogenic lines when fed the control M diet (dark grey) and when fed the V diet (light grey). Probes found to be differentially expressed only with the V diet (V diet ∩ M diet ^*C*^) were identified as potential molecular markers linked to plant-based diet utilisation, and were thus kept for further analysis (hatched area).

We compared the 3 isogenic lines amongst each other (R23h versus AB1h; R23h versus A22h; AB1h versus A22h) to identify genes which explain their divergent ability to use the V diet. The 6 different comparisons included: R23h-V versus AB1h-V and R23h-M *versus* AB1h-M; R23h-V *versus* A22h-V and R23h-M *versus* A22h-M; AB1h-V *versus* A22h-V and AB1h-M *versus* A22h-M. Two lists of genes differentially expressed were obtained per line comparison (M and V). However, given that these three isogenic lines display different genotypes, intrinsic differences are present among each of the lines. Differences in the expression of genes between lines could thus be a result of differences in genotype, and may have no relation to their ability to use the V diet. In order to exclude these genes from our results, the two lists of differentially expressed genes obtained per line comparison were compared. Probes that were found to be differentially expressed when fed both the M diet and the V diet were considered as intrinsic differences between genotypes, irrespective of the dietary treatment and were excluded from the analysis. Probes found to be differentially expressed only with the V diet (V diet ∩ M diet ^*C*^) were identified as potential molecular markers linked to plant-based diet utilisation, and were thus kept for further analysis (Fig 1). To interpret results, gene ontology (GO) enrichment analyses were performed using the functional annotation chart tool from the DAVID (Database for Annotation, Visualization and Integrated Discovery) bioinformatics resource, version 6.7 [32, 33]. Gene ontology (GO) enrichment analyses were carried out on the three lists of genes identified as potential molecular markers (Cut-off p-value<0.01; Benjamini<0.1). Swiss Prot identifiers were used as input against all Swiss protein identifiers of the array. Lastly, to help enrich the biological interpretation, the within line diet effect was assessed for genes previously kept, by calculating the fold change between fish fed the V diet *versus* the control M diet (R23h-V versus R23h-M; AB1h-V versus AB1h-M; A22h-V versus A22h-M) (Cut-off p-value = 0.05).

Concerning qPCR data, assays were carried out according to the MIQE (Minimum Information for Publication of Quantitative Real-Time PCR Experiments) standards [34]. The relative expression levels were calculated by a mathematical method based on the real-time PCR efficiencies [35]. The geometric average expression of GAPDH, 18S and EF1A was used for normalisation. Gene expression data were transformed with a logarithmic transformation. The normality and homogeneity of variance of residuals were also tested with the Shapiro-Wilk’s and Bartlett tests, respectively. Student t tests were performed to compare relative genes expression between genotypes when fed the M diet and then the V diet (Cut-off p-value = 0.05). Pearson’s correlations were calculated to quantify relationships between microarray and qPCR data.

### Ethical statement

Experimentation was conducted in the INRA experimental facilities (Peima facilities, Sizun, France) authorized for animal experimentation by the French veterinary service which is the competent authority (B 29-277-02). The experiments were in strict accordance with EU legal frameworks related to the protection of animals used for scientific research (Directive 2010/63/EU) and according to the National Guidelines for Animal Care of the French Ministry of Research (decree n°2013-118, february 1st, 2013). The scientist in charge of the experimentation received training and personal authorization (N°B64 10 003).

In agreement with ethical comittee “Comité d’Ethique Finistérien en Expérimentation Animale” (C2EA-74), the experiment reported here does not need approval by a specific ethical committee since it implies only classical rearing practices with all diets used in the experiment formulated to cover the nutritional requirements of Rainbow trout [8]. During the experiment, fish were daily monitored. If any clinical symptoms (i.e. morphological abnormality, restlessness or uncoordinated movements) were observed, fish were sedated by immersion in 2% benzocaine solution and then euthanized by immersion in a 6% benzocaine solution (anesthetic overdose) during 3 minutes.

## Results

### Biometric parameters

**Fig 2:**
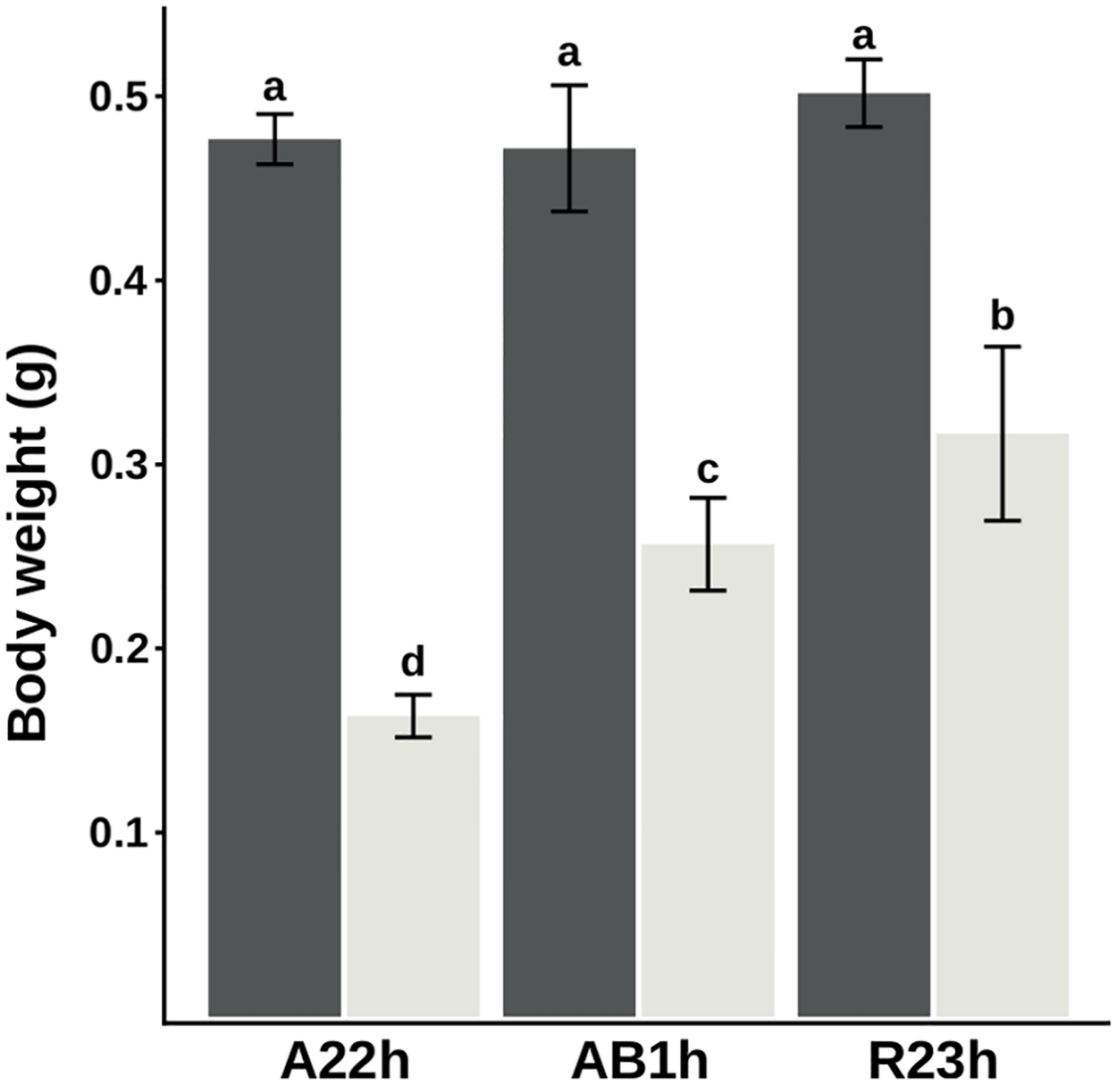
Mean body weights after 5 weeks of feeding. Final body weight (means±SEM) of the three isogenic lines fed either a diet M (control diet in dark grey) or V diet (in light grey) after 5 weeks of feeding. Two-way analysis of variance, followed by a Tukey post hoc test were carried out in order to assess effects of diet and genotype. Different letters mean significance between groups (Cut-off P-value<0.05).

Mean body weights of fry from each condition at the end of the feeding trial, i.e. after the first 5 weeks of feeding (74 dpf), are presented in Fig 2. While there were no significant differences in weight between isogenic lines that were fed the M diet (overall mean equal to 0.48±0.03g (mean±standard error of the mean), P-value>0.05), there were significant differences between isogenic lines when fed the V diet (P-value<0.01). As shown in Fig 2, R23h fed the V diet had the highest final weight in comparison to AB1h (-18%), and A22h (-50%). At the end of the 5 weeks trial, there was no effect of the line, nor the diet, on survival rates (95.7±2.46%).

### Microarray results

**Fig 3:**
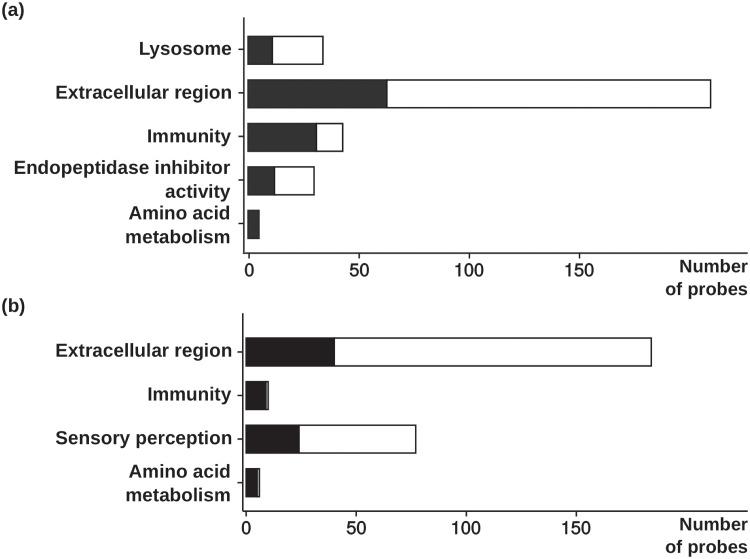
Gene ontology enrichment analysis results. Cluster with number of genes up (in black) and down-regulated (in white), issued from the gene ontology enrichment analysis using DAVID bioinformatics resource for the (a) R23h *versus* AB1h and (b) R23h *versus* A22h comparisons (Cut-off: p-value<0.01; Benjamini<0.1).

The numbers of genes differentially expressed between the three isogenic lines after 5 weeks of feeding with either the V diet or the M diet are summarised in Table 3. For the three line comparisons, 1740, 1834 and 246 probes were found to be differentially expressed only with the V diet, and were thus identified as potential molecular markers linked to feeding a plant-based diet (Supplementary file 2). Among those genes, 1279, 1253 and 186 have a swiss prot ID’s. To identify pathways potentially involved in plant-based diet utilisation, a gene ontology enrichment analysis was carried out only on potential molecular markers. For the three different comparisons, GO terms obtained were manually clustered in different categories of interest and are presented in Fig 3. The complete list of GO terms found significantly enriched is presented in the Supplementary file 3.

**Table 3:** Number of differentially expressed probes among conditions after limma t-test (corrected P-value≤0.05, Benjamini-Hotchberg).

	R23h *versus* AB1h	R23h *versus* A22h	AB1h *versus* A22h
M diet	2477	2135	4132
V diet	3403	3270	926
**V diet ∩ M diet ^*C*^**	**1740**	**1834**	**246**

GO categories related to extracellular region (210 and 184 probes, respectively), innate and adaptive immunity (42 and 10 probes, respectively), and amino acid metabolism (5 and 8 probes, respectively) were significantly enriched for R23h versus AB1h and R23h versus A22h comparisons. For R23h versus AB1h only, GO terms linked to lysosome and inhibition of endopeptidase activity were significantly enriched, and contained 35 probes and 30 probes, respectively. For R23h *versus* A22h comparisons, a cluster of significantly enriched gene ontology linked to neurological processes and perception contained 70 probes. Finally, no gene ontologies were found to be significantly enriched for AB1h versus A22h comparison after 5 weeks of feeding (Cut-off p-value<0.05, Benjamini Cut-off<0.1).

More precisely, the genes related to perception (Table 4) described here are linked to several sensory systems including vision, olfactory, gustatory and auditory perception and the majority were less expressed in R23h lines in comparison to A22h (57/70). Moreover, genes coding factors linked to feed intake regulation were also found. Both orexigenic (factors which positively regulate feed intake) and anorexigenic factors (which negatively regulate feed intake) were detected. Expression of the genes related to the different perception systems and feed intake were not affected in R23h by the V diet but were either up or down-regulated in A22h.

**Table 4:** Genes differentially expressed between the 3 isogenic lines related to perception.

Swiss Prot	Description	Genotype effect	Diet effect		
			R23h	AB1h	A22h
**Perception of smell/taste**					
P36510	UDP-glucuronosyltransferase 2A1 (Ugt2a1)	R23h <A22h	ns	ns	ns
P56373	P2X purinoceptor 3 (P2RX3)	R23h <A22h	ns	ns	up
Q3V3I2	Guanine nucleotide-binding protein G(t) subunit *α*-3 (GNAT3)	R23h <A22h	ns	ns	down
P23625	G protein *α*q subunit (G*α*q)	R23h <A22h	ns	ns	ns
**Vision**					
P43004	Excitatory amino acid transporter 2 (SLC1A2)	R23h <A22h	ns	up	up
Q9BGL2	Lecithin retinol acyltransferase (LRAT)	R23h <A22h	ns	ns	up
P35349	Metabotropic glutamate receptor 6 (Grm6)	R23h <A22h	ns	ns	up
P04181	Ornithine aminotransferase, mitochondrial (OAT)	R23h <A22h	ns	ns	up
P87368	Putative violet-sensitive opsin	R23h <A22h	ns	up	up
P87367	Red-sensitive opsin	R23h <A22h	up	up	up
Q9XT54	Retinal rod rhodopsin-sensitive cGMP 3’,5’-cyclic phosphodiesterase subunit delta (P2E6D)	R23h <A22h	ns	ns	up
Q9ZI275	Retinaldehyde-binding protein 1 (Rlbp1)	R23h <A22h	ns	up	up
Q8AXN9	Retinoid isomerohydrolase (RPE65)	R23h <A22h	ns	up	up
Q9YGX2	Retinoid isomerohydrolase (RPE65)	R23h <A22h	ns	ns	up
Q96NR8	Retinol dehydrogenase 12 (RDH12)	R23h <A22h	ns	ns	ns
P47804	RPE-retinal G protein-coupled receptor (RGR)	R23h <A22h	ns	up	up
**Sensory Perception of sound**					
O55225	Otogelin (Otog)	R23h <A22h	ns	ns	up
Q8NHW6	Otospiralin (OTOS)	R23h <A22h	down	up	up
P58743	Prestin (SLC26A5)	R23h >A22h	ns	ns	ns
Q810W9	Whirlin (Whrn)	R23h >A22h	ns	ns	ns
**Feed intake**					
P28566	5-hydroxytryptamine receptor 1E (Htr1E)	R23h >A22h	ns	down	down
P35563	5-hydroxytryptamine receptor 3A (Htr3A)	R23h >A22h	ns	ns	ns
Q9W6M9	Galanin (GAL)	R23h <A22h	ns	ns	up
Q9JLS8	Leptin receptor gene-related protein (Leprot)	R23h >A22h	ns	ns	up
P70579	Metabotropic glutamate receptor 8 (GRM8)	R23h <A22h	ns	ns	up
	Proopiomelanocortin B (PomCb)	R23h <A22h	ns	ns	ns

The genotype effect refers to genes whose expression differed between genotypes when they were fed with the V diet. The diet effect refers to the effect of the V diet for each isogenic line (“up” = gene was up-regulated when fed the V diet in comparison to the control diet; “down” = gene was down-regulated when fed the V diet in comparison to the control diet).

The genes related to the sulphur amino acid biological process were the betaine-homocysteine S-methyltransferase (BHMT1 and BHMT2), S-adenosylmethionine synthetase (Mat1a) and cysteine-*γ*-lyase (CGL), and were all more expressed in R23h when fed the V diet (Table 5). And while genes expressions were up-regulated in R23h and AB1h lines when fed the V diet, the diet did not affect expression of these genes in A22h.

**Table 5:** Genes differentially expressed between the 3 isogenic lines related to amino acid metabolism.

Swiss Prot	Description	Genotype effect	Diet effect		
			R23h	AB1h	A22h
Q68FT5	Betaine–homocysteine S-methyltransferase 2 (BHMT2)	R23h >AB1h, R23h >A22h	up	up	ns
Q32LQ4	Betaine–homocysteine S-methyltransferase 1 (BHMT1)	R23h >AB1h, R23h >A22h	up	up	ns
Q58DW2	Cystathionine gamma-lyase (CTH)	R23h >A22h	up	up	ns
P13444	S-adenosylmethionine synthetase isoform type-1 (Mat1)	R23h >A22h	up	up	ns

The genotype effect refers to genes whose expression differed between lines when they were fed with the V diet. The diet effect refers to the effect of the V diet for each isogenic line (“up” = gene was up-regulated when fed the V diet in comparison to the control diet; “down” = gene was down-regulated when fed the V diet in comparison to the control diet).

Concerning immunity, genes were related to the complement system, the major histocompatibility complex, and T and B cell regulation (Table 6). Most of these genes were expressed at a higher level in R23h in comparison to AB1h (31/43 genes) and A22h (9/10 genes). While expression of the majority of these genes was unaffected by the V diet in R23h, genes were either down or up-regulated in AB1h and A22h when fed the V diet.

**Table 6:** Genes differentially expressed between the three isogenic lines related to immunity.

Swiss Prot	Description	Genotype effect	Diet effect			Effect ANF
			R23h	AB1h	A22h	
**Complement**						
P98093	Complement C3 (Fragment) (C3)	R23h >AB1h	ns	ns	ns	down [57]
P04186	Complement factor B (CFB)	R23h >AB1h	ns	down	ns	
Q9BXR6	Complement factor H-related protein 5 (CFH)	R23h >AB1h	ns	ns	ns	
P20023	Complement receptor type 2 (CR2)	R23h >AB1h	ns	ns	up	
Q66S62	Mannose-binding protein C (MBL2)	R23h >AB1h	down	down	down	
Q5RBP8	Properdin (CFP)	R23h >AB1h	ns	down	down	up [54]
**T and B cell regulation**						
Q9NUV9	GTPase IMAP family member 4 (GIMAP4)	R23h >AB1h	ns	ns	ns	down [55]
Q8NHV1	GTPase IMAP family member 7 (GIMAP7)	R23h <A22h	ns	ns	up	up [55]
P06314	Ig kappa chain V-IV region B17 (IGKV4-1)	R23h >AB1h, R23h >A22h	up	ns	down	up [55]
P23735	Ig mu chain C region membrane-bound form	R23h <AB1h	down	ns	ns	up [55]
P23735	Ig mu chain C region membrane-bound form	R23h >A22h	ns	ns	down	up [55]
**Antigen processing and presentation**						
P14483	H-2 class II histocompatibility antigen, A *β* chain	R23h <A22h	ns	ns	ns	
P15979	Class I histocompatibility antigen, F10 *α* chain	R23h >A22h	ns	ns	ns	
P36372	Antigen peptide transporter 2 (Tap2)	R23h >A22h	ns	ns	ns	
Q9R233	Tapasin (Tapbp)	R23h >A22h	ns	ns	ns	
Q8VD31	Tapasin-related protein (Tapbpl)	R23h >A22h	ns	ns	ns	
Q8VCW4	Protein unc-93 homolog B1 (Unc93b1)	R23h >A22h	ns	down	ns	
Q9JJ22	Endoplasmic reticulum aminopeptidase 1 (Erap1)	R23h >AB1h	ns	down	ns	
P35737	Class II histocompatibility antigen, M *β*1 chain	R23h >AB1h	ns	down	ns	
Q95IT1	HLA class I histocompatibility antigen, *α* chain G	R23h >AB1h	ns	ns	ns	
P01897	H-2 class I histocompatibility antigen, L-D *α* chain	R23h >AB1h	ns	down	ns	
Q8HWB0	Major histocompatibility complex class I-related gene protein	R23h >AB1h	ns	down	ns	
P14483	H-2 class II histocompatibility antigen, A *β* chain	R23h >AB1h	ns	down	up	
P15981	SLA class II histocompatibility antigen, DQ haplotype D *α* chain	R23h >AB1h, R23h >A22h	ns	ns	ns	
P04223	H-2 class I histocompatibility antigen, K-K *α*chain	R23h >AB1h, R23h >A22h	ns	down	ns	

The genotype effect refers to genes whose expression differed between lines when they were fed with the V diet. The diet effect refers to the effect of the V diet for each isogenic line (“up” = gene was up-regulated when fed the V diet in comparison to the control diet; “down” = gene was down-regulated when fed the V diet in comparison to the control diet) Effect of ANF refers to the known effect of ANF, according to literature.

In addition, some genes previously found as enteropathy markers were also retrieved and were listed in Table 7. Among those genes, some were related to the epithelial barrier, the activation of the T and B cell functions via the up-regulation of the GTPase IMAP family member, or the NF-*κ*-B inhibitor*α* pathway.

**Table 7:** Genes, differentially expressed between the three isogenic lines, previously identified as enteropathy markers.

Swiss Prot	Description	Genotype effect	Diet effect			Effect ANF
			R23h	AB1h	A22h	
Q8HZM6	Annexin A1 (ANXA1)	R23h <AB1h, R23 <A22h	ns	ns	up	up [53, 54]
P24801	Annexin A2-B (anxa2-b)	R23 <A22h	ns	ns	ns	up [55]
Q8HYP5	C-C motif chemokine 21 (CCL21)	R23h >AB1h	ns	down	ns	down [54]
P53569	CCAAT/enhancer-binding protein zeta (Cebpz)	R23h >AB1h	ns	down	ns	up [53, 54]
Q56JX9	Fatty acid-binding protein,intestinal (FBP2)	R23h <AB1h	ns	up	ns	up [54, 56]
P13284	Gamma-interferon-inducible lysosomal thiol reductase (IFI30)	R23h >A22h	ns	ns	down	down [54]
Q9BDB7	Interferon-induced protein 44-like (Ifi44l)	R23h >AB1h	ns	ns	down	down [54, 55]
O62644	Leukocyte cell-derived chemotaxin-2 (LECT2)	R23h <AB1h, R23 <A22h	ns	ns	up	up [53]
Q1JPB0	Leukocyte elastase inhibitor (SERPINB1)	R23 <AB1h	ns	ns	ns	up [54, 55]
O60449	Lymphocyte antigen 75 (LY75)	R23h >AB1h	ns	ns	ns	down [53]
P25963	NF-kappa-B inhibitor*α* (NFKBIA)	R23h <AB1h	ns	up	ns	up [54]
**Epithelial barrier**						
Q9Y251	Heparanase (HSPE)	AB1h >A22 h	ns	up	ns	up [55]
P14780	Matrix metalloproteinase-9 (MMP9)	R23 <A22h	up	up	up	up [54]
Q9TTY1	Metalloproteinase inhibitor 2 (TIMP2)	R23 <A22h	ns	down	up	up [54]
Q02817	Mucin-2 (Muc2)	R23h <A22h	ns	ns	ns	up [55]
Q04666	Transcription factor HES-1 (Hes1)	AB1h >A22h	ns	ns	down	up [55]

The genotype effect refers to genes whose expression differed between lines when they were fed with the V diet. The diet effect refers to the effect of the V diet for each isogenic line (“up” = gene was up-regulated when fed the V diet in comparison to the control diet; “down” = gene was down-regulated when fed the V diet in comparison to the control diet) Effect of ANF refers to the known effect of ANF, according to literature.

The important number of genes related to the extracellular component (GO cellular component category) had highly heterogeneous functions.

### qPCR

**Fig 4:**
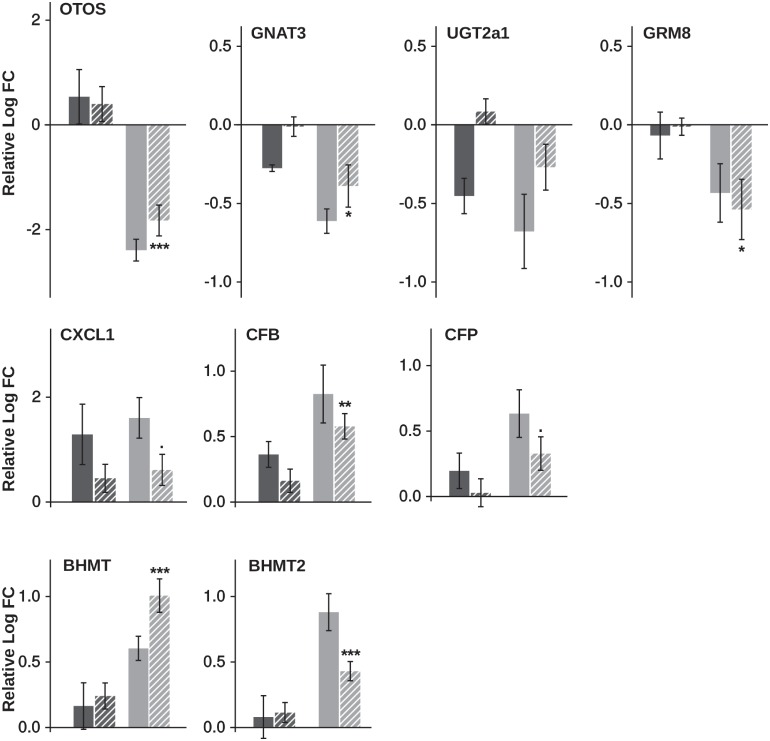
qPCR results. Fold changes obtained by microarray (fill) and by qPCR (hatched) when fed the M diet (dark grey) or the V diet (light grey) for genes involved in sensory perception (R23h *versus* A22h), immunity (R23h *versus* AB1h) and for amino acid metabolism (R23h *versus* A22h).

In order to check microarray results, 9 genes found differentially expressed through the transcriptome analysis and linked to immunity, perception, and metabolism were tested by real-time qPCR. Fold changes obtained with both microarray and qPCR are presented in Fig 4. For all genes tested, relative expressions were not significantly different between genotypes when fish were fed the M diet, as expected. In contrast, expressions were significantly different between genotypes fed with the V diet (Cut-off p-value<0.05 and Cut-off p-value<0.1 for CXCL1 and CFP). Fold changes obtained by qPCR and microarray were in agreement, therefore validating transcriptomic results. Coefficients of correlation showed that qPCR and microarray results were correlated for 8/9 genes (Pearson correlation coefficient>0.60 and p-value<0.01) (Table 8).

**Table 8:** Correlation between gene expression patterns obtained through real-time PCR and microarray approaches.

Gene		Correlation Coefficient	P-value
**Amino acid metabolism**			
BHMT	Betaine-homocysteine S-methyltransferase 1	0.71	2.78E-06
BHMT2	Betaine-homocysteine S-methyltransferase 2	0.69	7.28E-06
**Immunity**			
CFB	Complement factor B	0.61	1.39E-04
CFP	Properdin	0.51	2.26E-03
CXCL1	Growth-regulated *α* protein	0.71	4.45E-06
**Sensory Perception**			
OTOS	Otospiralin	0.92	7.11E-15
Ugt2a1	UDP-glucuronosyltransferase 2A1	0.62	1.04E-04
GNAT3	Guanine nucleotide-binding protein G(t) subunit *α*-3	0.78	4.80E-08
GRM8	Metabotropic glutamate receptor 8	0.50	2.40E-03

Coefficient correlation were obtained with a Pearson test.

## Discussion

In order to identify genes which discriminate trout genotypes in their ability to grow on a full plant-based diet at an early-stage, which is a critical step in their future capacity to grow with such diet [24], we compared transcriptomic profiles of three RBT isogenic lines, which differ in their growth response when fed a plant-based diet. Transcriptomic analyses are considered appropriate to tackle questions related to nutrition without any *a priori* hypothesis.

Effects of plant-based diets have been largely studied, but only few transcriptomic studies have analysed the effect of fish genetic background. Overall, these studies indicated only a limited number of genes to be differentially expressed according to the genotype in RBT [20, 21], in salmon [11] or in sea bass [14]. In the present experiment, we found a high number of genes differentially expressed between genotypes, with a cut-off p-value corrected for multiple testing, controlling thus the number of false positives. This is probably due to the reduced variability in gene expressions within each isogenic line, as fish share the same genotype. This enhances the capacity to detect differences between lines, confirming the benefit of using this relevant biological material.

### Molecular markers linked to the reduction of the feed intake

One of the first suggested reason to explain the lower growth when fish are fed plant-based diets is a disturbance of fish feeding behaviour and a lower feed intake [7, 36, 37]. Two main hypotheses were put forward to explain the perturbation of feeding behaviour with plant-based diets [37]. The first one is a change in palatability that could provoke an aversion to such a diet, mainly due to differences in AA profiles [7, 38]. The other possible explanation is a negative feedback due to the nutritional quality of diets, which could affect appetite [37].

In the present study, even though diets meet nutrient requirements and are supplemented with an attractant mix like, the two experimental diets differ in their AA profiles. The major difference lies on glutamic acid content which is twice higher in the V diet than in the M diet. This AA has been shown to reduce locomotor activity related to feeding behaviour [38] and could therefore explain the lower growth observed when fish were fed the plant-based diet, regardless of their genotype background (Fig 2).

When we investigated the effect of the genotype, the transcriptomic analysis has allowed the detection of a genetic variability in the expression of genes related to perception and regulation of feed intake between R23h and A22h fish at an early-stage. Results have been confirmed by qPCR for the genes tested (Fig 4).

#### Perception

All the genes linked to perception were under expressed in R23h, and except the red-sensitive opsin, the expression of those genes was not affected by the V diet in the R23h fish but was significantly up-regulated in A22h fish fed the V diet. Noteworthy, the expression of several of these genes (e.g. Opsin, Retinaldehyde-binding protein, GRM8) was also altered in the brain of juvenile RBT following a plant-based diet feeding period at the fry stage, suggesting a long-term programming effect of early plant-based diet exposure on pathways of sensory perception [25].

Olfaction and vision are of primary importance to trigger the feeding behaviour in RBT [38, 39]. These differences in genes expression of these two components may reflect differences in the acceptance of the V diet between genotypes. We present, for the first time, a list of genes that could potentially affect the acceptance of a plant-based diet during the first weeks after first-feeding. Most of these genes have not been characterised in fish, and further investigations are needed to determine their specific roles in the acceptance of plant-based diets in RBT.

#### Appetite-regulating peptides

Variations in mRNA expression of genes encoding molecules involved in the control of feed intake were also found in the genotypes fed the V diet. Neuropeptide expression, which presents a high variation during and after meal time, is impacted by the quantity of feed ingested by fish. As outlined by Balasubramanian *et al.*, changes in their expression level may indicate downstream effects caused by differences in feed intake [25]. But variations in the expression or regulation of neuropeptides can also directly induce different feed intake among the genotypes. In this case, higher expression of orexigenic factors in R23h in comparison to A22h might reflect higher appetite for the plant-based diet. In our study, two serotonins (Htr3a and Htr1e) were more expressed in R23h in comparison to A22h when fed the V diet. Hayes *et al.* [40] found that Htr3a receptor positively regulates feed intake in rats. GRM8 can also influence NPY [41, 42], known to stimulate feed intake, but its role is not yet well understood. In contrast, the PomC-b gene which is the precursor peptide for the melanin-concentrating hormones (MSH) which negatively regulates feed intake [43], was less expressed in R23h than in the A22h lines when fed the V diet. A particular attention should therefore be given to these genes, which might be molecular markers related to feed intake regulation.

To conclude, besides the known effect of plant-based diets on feed intake [7, 38], it could exists differences of feed consumption among RBT genotypes when fed such a diet. The genetic variability in the expression of genes related to the V diet intake, revealed by our transcriptomic analysis, could indeed mirror differences between genotypes in tolerance to plant-based diets, either through perception mechanisms or through direct effects on feeding motivation. Fish from the R23h could have a lower aversion to components present in the V diet or a higher tolerance to the modified AA profile, impacting thus their feed intake to a lesser extent. On the contrary, the feeding behaviour of fish with the lowest growth rate (A22h) could be drastically impacted by the plant-based diet.

Differences in the V diet intake could be the first step distinguishing RBT genotypes on their ability to grow with a plant-based diet. These results are in line with previous data obtained after a long-term feeding trial, where different feed intakes were recorded for these same isogenic lines [24]. Feed consumption among RBT would be very useful to confirm these results. However, an accurate measure of feed intake is not possible at early life stages of fish.

### Few genes related to digestion, absorption and metabolism

In addition to a possible lower feed intake, a lower feed efficiency is also known to impact growth with plant-based diets [5, 9–15, 44]. Lower feed efficency could stem from the impairment of digestion and absorption [44], and/or the alteration of metabolism [5, 9–15].

Impairment of the steps of digestion and absorption might be due to the presence of anti-nutritionnal factors such as non-starch polysaccharides. Presence of these compounds in plant-based diets are known to alter the expression of various genes involved in nutrients absorption [45]. However, in our study, none of these genes were differentially expressed among the three lines of RBT studied and no GO linked to these processes were found. Whereas the diminution of growth rate when fish were fed the plant-based diet (Fig 2) could be due to the impairment of the digestion and the absorption, our results suggested that no differences in the capacity to digest or absorb nutrients explain differences in growth observed among genotypes fed the V diet at an early stage. This could be because the ingredients of the V diet were choosen to limit non starch polysaccharides content of this diet.

Regarding metabolism, plant-based diets are known to highly affect various pathways due to differences in the AA profile, the FA profiles but also the content of minerals or vitamins [5, 9–15].

However, only differences in the sulfur-containing AA metabolism were revealed by our analysis among the three genotypes when fed the V diet. Expression of genes encoding for enzymes from these three pathways (BHMT1, BHMT2, Mat1a, CGL) were up-regulated by the V diet in the two genotypes having the best growth rates (R23h and AB1h). Results have been confirmed for the two genes tested (BHMT1 and BHMT2) by qPCR (Fig 4).

Differences observed in gene expression could revealed differences in the capacity of RBT genotypes to cope with the different AA profile in the plant-based diets (Table 1). Sulfur-containing AA metabolism is crucial in fish as methionine donates a methyl group to methylate DNA, proteins, lipids, and other intracellular metabolites that lead to homocysteine formation (transmethylation pathway). Homocysteine can then either be recycled to methionine (remethylation pathway), or engaged in the formation of cysteine and taurine (transsulfuration pathway) [46, 47]. Due to the importance of this pathway, these differences could thus have a vast impact on other pathways, and explained thus the difference of growth observed among genotypes. For instance, results obtained in the present study possibly indicated either a higher taurine biosynthesis in R23h in comparison to A22h, or a higher requirement for taurine in A22h genotype which may contribute to the growth differences. This result confirms the interest of paying attention to the level of taurine in plant-based diets, in addition to that of methionine as it is known that taurine supplementation improved RBT growth [48].

### Immunity: R23h line seems to be less affected by the plant-based diet

Finally, several genes linked to immunity were identified to vary among genotypes in our analysis of fry transcriptome. Results have been confirmed for two genes amongst the three tested (Fig 4). Due to the whole genome duplication that occurred in salmonids [27], validating transcriptomic data by qPCR studies are often challenging due to the presence of duplicated and highly similar genes whose transcripts might be differentially regulated [28].

The results obtained in the present study demonstrated the existence of a genetic variability in the expression of genes related to immunity at an early-stage. This result corroborates a previous transcriptomic analysis which also revealed a differential expression of some immunity genes between two families of European sea bass differing in their growth response to a plant-based diet [14]. Overall, the effect of the diet seems negligible in the R23h line as expression of most genes related to immunity was unaffected by the V diet. In contrast, the majority of these genes were down-regulated in the two other genotypes when fed the V diet. R23h fish could be less affected by dietary factors and fish from these genotypes could spend less energy on modulation of the immune function, improving thus its growth.

#### Lack of *ω*-3 LC-PUFA

FO and vegetable oils differ greatly in their FA profile, and this affects gene expression related to immunity [10, 11, 16] and immunity parameters by altering immune cell phospholipids and/or eicosanoid production, key hormones in immune response derived from FA [49]. While *ω*-3 LC-PUFA such as EPA (eicosapentaenoic acid), abundant the M diet, are precursors of prostaglandins and leukotrienes, known to have anti-inflammatory action in mammals [50, 51], the V diet is rich in omega-9 and omega-6 LC-PUFA, which produce mainly pro-inflammatory molecules [52].

No genes related to the lipid metabolism were however found differentially expressed among the three genotypes when fish were fed the V diet. But as the use of whole fry could potentially hide some differences in gene expression [28], we hypothesize that fish with higher growth rates (R23h and AB1h) could have higher capacity to biosynthesized *ω*-3 LC-PUFA and might thus be less susceptible to their lack in the plant-based diet (Table 1). This hypothesis is reinforced by data obtained in liver of the three same genotypes after 6 months of feeding where the gene coding for the FA desaturase and elongase implicated in biosynthesized of these LC-PUFA was more expressed in the AB1h and R23h fish (unpublished data).

#### Presence of ANF

Numerous studies have shown that anti-nutritional factors (ANF) present in vegetable meals, may cause gut inflammation (enteritis), characterised by an intestinal dysfunction. Presence of ANF leads to a rapid alteration of liver and intestine transcriptome profiles [53–59], even when diets were carefully formulated to diminish their negative effects [13].

Although, effects of ANF has not been clearly demonstrated at early-stage in Atlantic salmon fry fed a diet containing 16.7% soybean meal [55], we could also hypothesize that modifications in the transcriptomic profiles of AB1h and A22h fish might indicate subtle changes in biological processes. These two genotypes might be affected by ANF, while R23h fish might be less susceptible to these factors. Expression of some of the previously identified enteropathy markers indicated indeed that AB1h and A22h fish may be affected by ANF (Tables 6 and 7). Among those genes, some genes were involved in the epithelial barrier [54, 55], the activation of the T and B cell functions via the up-regulation of the GTPase IMAP family member 7, and the major histocompatibility complex, which plays a crucial role by presenting antigenic peptides to T cells [60]. Moreover, annexins which are usually highly up-regulated during enteritis, contributing to the intestinal resistance due to their anti-inflammatory properties [53–55], were also up-regulated in the A22h line. Other markers such as the fatty acid-binding protein or the NF-*κ*-B inhibitor*α* were also up-regulated in AB1h, as expected during enteritis [54, 56].

Expression of some of the previously identified enteropathy markers was however regulated opposite to what would be expected during enteritis in AB1h or A22h (Ig mu chain C, Hes-1, Cebpz), or was also affected in the R23h line (IGKV4-1, MMP-9). And, as both FO and FM were replaced, expression of genes related to immunity could be also affected by the interaction between ANFs and FA (Complement system for example). Further analyses after a long-term feeding (transcriptomic and histological analysis of the intestine) are needed to gather information on how fish from these three lines were affected by those different factors to confirm the hypotheses.

#### Other factors

In addition to these two well known issues, other less studied factors could also impact immunity when fish are fed a plant-based diet. While AA play also a central role in immunity [61], the AA profile of the V diet differ significantly from the one of the M diet. Among the most contrasted differences, the glutamic acid content, an AA known to have a role in the intestinal health [61], is highly different in the two experimental diets.

Finally, mineral and vitamins are essential components in fish immunity [62]. Although the V diet is supplemented with a mix of mineral and vitamins to meet the known RBT nutrient requirements, it could not be excluded that small differences exist between the two experimental diets.

Along with a higher capacity to biosynthesize LC-PUFA, it could be assumed that the variability of expression could also arise from different capacity to cope an imbalanced AA profile or a lack of some mineral and/or vitamins. And it could be the case for R23h fish, explaining thus their higher growth when fed the V diet.

## Conclusion

Isogenic lines constitute a relevant biological material in this study as the reduced variability within each line enhances the statistical power to detect differences among groups. The transcriptomic approach, without *a priori* hypothesis, allowed us to set up a list of molecular markers which may play a role in the acceptance and utilisation of plant-based diets at an early stage, known as a critical rearing phase [22, 24]. Only analysis on whole fry are feasible at this highly important stage, possibly hiding some effects on specific organs [28] and further analyses will be needed on specific organs to strengthen our findings.

Nevertheless, we were able to identify an important number of molecular markers linked to the utilisation of a plant-based diet. Moreover, the comparison of three isogenic lines with two genotypes performing better with plant-based diets (R23h and AB1h) than the third one (A22h) reveals different pathways, showing that there are different possible strategies to optimise utilisation of such diets.

Several of these genes had been previously identified in other studies and we confirm their potential role in the utilisation of a plant-based diet (immunity, sulfur-containing AA metabolism). In addition, we found new genes, several of which are linked to sensory perception and appetite regulation. Pathways linked to vision, olfaction and feed intake regulation had previously been mentioned in a transcriptomic analysis that studied the nutritional programming of plant-based diets acceptance in RBT [25]. These convergent data strongly suggest that sensory perception is a key determinant of the acceptance of plant-based diets at an early stage. Studying sensory perception mechanisms and the regulation of feed intake by dietary factors should help improve the formulation of plant-based diets, thus improving performance of fish fed diets devoid of marine ingredients.

## Supporting information

S1 File Sequence of primers used for gene expression analysis by real-time PCR. (XLSX)

S2 File Fold changes and p-value associated with each gene found differentially expressed among the three genotypes, identified as potential molecular markers linked to plant-based diets utilisation. (XLSX)

S3 File GO terms found significantly enriched (Cut-off: p-value<0.01; Benjamini<0.1). (XLSX)
